# Salmonellosis Among Children Aged 0–14 Years in Greece over the Period 2005–2024: Descriptive Analysis of Surveillance Data from the Mandatory Notification System

**DOI:** 10.3390/microorganisms14040743

**Published:** 2026-03-26

**Authors:** Lida Politi, Theologia Sideroglou, Eleni Triantafyllou, Georgia Mandilara, Anthi Chrysostomou, Kassiani Mellou, Theano Georgakopoulou, Karolina Akinosoglou

**Affiliations:** 1 Department of Microbial Resistance and Antimicrobial Use, Directorate of Tackling Antimicrobial Resistance and Healthcare-Associated Infections, National Public Health Organization, 15123 Athens, Greece; l.politi@eody.gov.gr; 2 School of Science and Technology, Hellenic Open University, 26331 Patras, Greece; 3 Department of Foodborne/Waterborne Diseases and Food Safety, Directorate of Epidemiological Surveillance and Prevention of Communicable Diseases, National Public Health Organization, 15123 Athens, Greece; t.sideroglou@eody.gov.gr (T.S.);; 4 Department of Surveillance Systems Coordination, Directorate of Epidemiological Surveillance and Prevention of Communicable Diseases, National Public Health Organization, 15123 Athens, Greece; e.triantafyllou@eody.gov.gr; 5 National Reference Centre for Salmonella, School of Public Health, University of West Attica, 11521 Athens, Greece; gmandilara@uniwa.gr; 6 Directorate of Data Management, General Directorate of Information Technology, National Public Health Organization, 15123 Athens, Greece; k.mellou@eody.gov.gr; 7 Directorate of Epidemiological Surveillance & Prevention of Communicable Diseases, General Directorate of Epidemiological Surveillance, National Public Health Organization, 15123 Athens, Greece; 8 Faculty of Medicine, University of Patras, 26504 Patras, Greece; 9 Department of Internal Medicine and Infectious Diseases, University General Hospital of Patras, 26504 Patras, Greece

**Keywords:** salmonellosis, children, *Salmonella* Enteritidis, *Salmonella* Typhimurium, Greece

## Abstract

Foodborne diseases remain a major public health challenge. Among them, salmonellosis is one of the most frequently reported illnesses, associated with clusters and outbreaks and with considerable morbidity, potentially severe in vulnerable populations. Children are more susceptible due to biological, behavioral, and dietary factors. This study aimed to summarize and describe national surveillance data from the Mandatory Notification System, combined with serotyping data, on reported salmonellosis cases in Greece during the period 2005–2024, with a focus on children aged 0–14 years. During the study period, a total of 7340 salmonellosis cases were reported among children aged 0–14 years. Notification rates declined gradually until 2021, followed by an increase through 2024. The mean annual notification rate was 23.0 cases per 100,000 population, with the highest incidence observed among children aged 0–4 years. A clear seasonal pattern was observed, with a peak during summer months, alongside notable geographical variation. The most frequently identified serovars were *Salmonella* Enteritidis and *Salmonella* Typhimurium. These findings indicate that salmonellosis remains a public health concern in the pediatric population, highlighting the need for enhanced surveillance, improved food hygiene practices, and targeted prevention strategies to reduce disease burden.

## 1. Introduction

*Salmonella* spp. is one of the main causative agents of foodborne infections, capable of causing foodborne outbreaks on international, national, and local levels [1,2,3]. In the European Union, salmonellosis is the second most frequently reported cause of gastrointestinal infection; the five most frequent *Salmonella* serovars in the European Union, in descending order, are Enteritidis, Typhimurium, monophasic Typhimurium, Infantis, and Newport [4]. Human infection by *Salmonella* strains is transmitted via the consumption of contaminated foods of animal origin (mainly eggs, meat, poultry, and milk). Other routes include the consumption of inadequately cooked meat, improper food handling, and consumption of unpasteurized dairy products and seafood. Fresh products, such as vegetables and fruit, may also be implicated in transmission, especially if there is manure or irrigation water contamination [5,6,7,8,9].

In most cases, infections caused by *Salmonella* spp. are self-limiting and do not require antimicrobial treatment. However, antimicrobial therapy is essential and can be lifesaving in invasive salmonellosis, which primarily affects individuals with specific risk factors, including young children, the elderly, and the immunocompromised [10,11,12,13,14,15]. Immunocompromised children—such as those with hemoglobin disorders, HIV infection, malignancies, or other underlying conditions—are at particularly high risk of severe disease and mortality due to complications [16].

Salmonellosis in children constitutes a major public health concern, especially in low- and middle-income countries [17,18]. This increased vulnerability can be attributed to immaturity of the immune system, particularities of the intestinal microbiota, as well as behaviors that favor exposure to pathogenic microorganisms [19,20]. Several retrospective reviews and studies have shown that this age group, particularly infants, is the most susceptible to non-typhoidal salmonellosis groups [13,21,22,23,24,25]. Clinically salmonellosis develops mainly as an acute gastrointestinal syndrome, with fever, abdominal pain, diarrhea, and, in severe cases, dehydration, hemorrhagic colitis, or even sepsis [26,27].

In Greece, the responsible authority for salmonellosis surveillance is the National Public Health Organization (EODY). There are two parallel surveillance systems for salmonellosis: the Mandatory Notification System (MNS) and the laboratory surveillance system through the National Reference Centre for Salmonella–Shigella (NSSRC). This system is voluntary, but universal, including all clinical microbiology laboratories across the country which are advised to submit isolates to the NSSRC for further typing. Data concerning cases and clusters of salmonellosis are collected during investigations and entered into a dedicated database at the Department of Foodborne/Waterborne Diseases and Food Safety of EODY [28].

Since 2010, efforts have been made to improve case notification by physicians due to existing underreporting, through feedback to hospitals, informational publications on the importance of notification, and other measures [13,29]. According to national surveillance data, during the period 2004–2020, a total of 10,707 salmonellosis cases were reported, with a mean annual notification rate of 5.7 cases per 100,000 population, while the most frequently reported non-typhoidal *Salmonella* serovars in Greece were *Salmonella* Enteritidis (*S.* Enteritidis) and *Salmonella* Typhimurium (*S.* Typhimurium) [28,30]. This study analyzed confirmed pediatric salmonellosis cases in Greece (2005–2024) to describe temporal, geographical, demographic, and serovar patterns, while inferential analysis was not performed.

## 2. Materials and Methods

### 2.1. Study Design and Data Sources

This was a retrospective epidemiological study of confirmed salmonellosis cases (children) aged 0–14 years who were residents of or travelers to Greece during the period 2005–2024. The study utilized the MNS case database. The MNS database includes data reported to EODY and derived from salmonellosis notification forms. In Greece, it is mandatory for all physicians, in both the public and private sectors, to notify cases of salmonellosis to local public health authorities and to EODY. Cases are classified according to the 2018 European Commission (EC) case definitions (Commission Decision 2018/945/EC). Notification forms include demographic characteristics (name, sex, date of birth, place of residence), clinical symptoms, laboratory data, and possible epidemiological linkage to other cases. At the same time, isolated *Salmonella* strains are sent to the NSSRC accompanied by a brief form containing the patient’s name, demographic data, and information about the specimen. These data are also transmitted to the ECDC (EpiPulse Cases and former European Surveillance System—TESSy platforms). During the study period, the surveillance system evolved operationally. In the early 2010s, notifications were largely transmitted via fax-based workflows, which were later progressively supplemented by strengthened laboratory confirmation and integration of reference laboratory isolates. Since 2020, encrypted electronic notification has formally replaced fax-based transmission, aiming to improve completeness and timeliness of reporting.

Data were retrieved from the database of the Directorate of Epidemiological Surveillance and Interventions for Infectious Diseases (DESIID) of EODY. Data on reported salmonellosis cases from the NSSRC had already been collected for the period 2005–2024 regarding *Salmonella* serotyping and had been incorporated into the MNS database. Population data were obtained from the Hellenic Statistical Authority (ELSTAT), based on the estimated population and migration flows of the country, by sex and five-year age groups (https://www.statistics.gr/el/home, accessed on 1 February 2026) and used as denominators.

Domestic cases were included in the study, whereas imported cases (namely cases that were not infected in Greece but were diagnosed and reported while in Greece) were excluded from the analysis.

#### Definitions

A confirmed case of salmonellosis is defined an individual, meeting the clinical criteria (at least one of the following: diarrhea, fever, abdominal pain, vomiting) and the laboratory criteria (at least one of the following: isolation of *Salmonella* (other than *S.* Typhi or *S.* Paratyphi) in a clinical specimen, detection of nucleic acid from *Salmonella* (other than *S.* Typhi or *S.* Paratyphi) in a clinical specimen) in accordance with the EC case definition [31].

A probable case of salmonellosis is an individual, meeting the clinical criteria with an epidemiological link (at least one of the following five epidemiological links: human to human transmission, exposure to a common source, animal to human transmission, exposure to contaminated food/drinking water, environmental exposure).

An epidemiological link of a case is defined as the presence of at least one person with similar symptoms among the close contacts.

### 2.2. Data Analysis

Descriptive data analysis was performed using the Stata 19 statistical package (StataCorp LLC, College Station, TX, USA) and MS Excel, following data cleaning. Measures of frequency (mean annual notification rate, standard deviation) by age, age group, and sex were used to describe cases among children aged 0–14 years in Greece for the period 2005–2024. Characteristics of cases (sex, age, age group, region of case notification, and temporal distribution for the description of seasonality) were presented in tables, maps, and graphs. Variables related to epidemiological linkage, travel history, and reported exposure settings were analyzed descriptively and not evaluated as independent or causal risk factors. Depending on data availability, proportions of cases were calculated according to clinical manifestations, hospitalization, travel history, and available epidemiological linkage. Associations between hospitalization and categorical variables (sex and age group) were assessed using chi-square (χ^2^) tests. Annual total numbers and annual notification rates were calculated at the national level, as well as the mean total number of cases per month of symptom onset for the period 2005–2024. The geographical distribution of cases was assessed by calculating mean notification rates per 100,000 population per region for the period 2005–2024, to identify potential increasing or decreasing prevalence patterns. Available epidemiological data were used to describe temporal changes in notification rates during the study period, by serovar and age groups following stratification (0–4 years, 5–9 years, 10–14 years). The ECDC EMMa Map Maker online tool (European Centre for Disease Prevention and Control, Stockholm, Sweden; https://www.ecdc.europa.eu/en, accessed on 1 February 2026) was used for the creation of incidence maps [32].

### 2.3. Personal Data Protection

All information remained confidential in accordance with the rules of the GDPR and Greek legislation (Law 4624/2019). Already collected data were anonymized and sent to EODY, the competent authority for the surveillance of communicable diseases under Greek Law. Subsequently, data were entered into the integrated database at the EODY’s premises, processed and analyzed according to national and European Union laws. Approval of the implementation of the protocol and publication in a scientific journal was granted by the Ethics Committee of EODY, following submission of the study protocol (approval protocol number: ΕΞΕ-ΚΠ 17571/17 September 2025).

## 3. Results

### 3.1. Population Overview

In Greece, during the period 2005–2024, a total of 7340 confirmed salmonellosis cases were reported among children aged 0–14 years, while 7154 confirmed salmonellosis cases were used for analysis after excluding imported cases. 5973 (84%) were reported by public hospitals, 1070 (15%) by private hospitals, and the remaining (1%) by private diagnostic laboratories, health centers, and private physicians. The median age of cases was 3 years (IQR: 6 years). Of these, 3880 cases (54%) were male children, 3267 (46%) were female, while for 7 cases (0.1%) sex was not reported.

For male cases, the median age was 3 years (IQR: 6 years), while for female cases the median age was also 3 years (IQR: 6 years). Further analysis by age group showed that 61% of all recorded cases belonged to the 0–4-year age group, with a median age of 1 year (IQR: 3, 53% male cases). This was followed by the 5–9-year age group (26%), with a median age of 7 years (IQR: 3, 55% male cases), and the 10–14-year age group (13.0%), with a median age of 12 years (IQR: 3, 59% male cases).

Of all reported cases, 6002 (84%) were Greek nationals, 480 (6%) were migrants, 175 (2%) were tourists, 15 (0.1%) were refugees, while for 497 cases (8%) no data were available. Regarding the presence of special population groups, only 5% of reported cases belonged to such groups; among those, 4% belonged to the Greek Roma population.

During the period 2005–2024, the mean annual notification rate of salmonellosis among individuals aged < 15 years was 23.0 cases per 100,000 population (Table 1). Analysis by five-year age subgroups (0–4 years, 5–9 years, 10–14 years) showed that salmonellosis was most frequent in the 0–4-year age group, where the mean annual notification rate was 49.9 cases per 100,000 population (Table 2).

### 3.2. Exposure-Related Factors

During the same period, 1080 reported cases (15%) indicated an epidemiological link with another case. Additionally, 175 cases (2.4%) were tourists, and 186 cases (2.5%) reported recent travel history abroad within the incubation period. Of these, 54% belonged to the 0–4-year age group, 27% to the 5–9-year age group, and 19% to the 10–14-year age group. Those with recent travel history abroad within the incubation period were excluded from the downstream analysis, as they were considered imported cases.

Twenty-eight percent of cases reported attendance in a school setting. Among these, 29% belonged to the 0–4-year age group, 44% to the 5–9-year age group, and 27% to the 10–14-year age group.

### 3.3. Hospitalization and Clinical Severity

During the period 2005–2024, 6733 children aged 0–14 years (92%) reported clinical manifestations of salmonellosis. Enteritis (81%) and enteric fever (17%) were the most frequently reported, followed by dysentery (9%) and sepsis (1%). This distribution was maintained when analysis was conducted by age group. Clinical symptoms by age and sex are presented in Table 3.

Overall, 6261 cases (88%) were hospitalized; among them, 54% were male children. Further analysis by age group showed that 60% of hospitalized cases were 0–4 years old (54% male), 26% were 5–9 years old (55% male), and 14% were 10–14 years old (58% male). Analysis showed that there was no statistically significant association between hospitalization and sex (*p* = 0.9314) of the child. Also, there was no statistically significant association between hospitalization and age group (*p* = 0.4966) of the child.

Invasive infections (bacteremia, sepsis) were recorded in 2% of cases, while other severe clinical manifestations (renal failure, myocarditis, etc.) were recorded at rates < 1%. Among all cases, 65% reported recovery at the time of notification, while 25% reported ongoing illness. Death was the outcome in three cases (0.04%), two of whom were male children. All three fatal cases belonged to the 5–9-year age group. None belonged to a special population group, or epidemiological link to a confirmed case in their immediate environment, and only two had been hospitalized prior to death.

### 3.4. Temporal Notification and Hospitalization Rate

**Figure 1:**
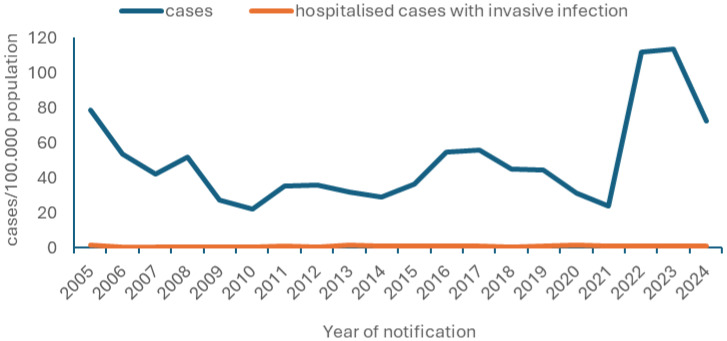
Time distribution of salmonellosis notification rates and of salmonellosis hospitalized case (with invasive infection) notification rates, MNS, Greece, 2005–2024.

During the period 2005–2024, a gradual decline in disease incidence among children aged < 15 years was observed (Figure 1) until 2021, after which an increase was noted. The same pattern was observed following age stratification (Supplementary Figure S1). The post-2021 increase continued until 2023, followed by a subsequent decline. The temporal distribution of salmonellosis notification rates and cases requiring hospitalization due to invasive infection among children aged 0–14 years during 2005–2024 is shown in Figure 1. Table 4 presents the total number of hospitalized salmonellosis cases among children aged 0–14 years per year of notification and age group.

### 3.5. Seasonality and Geographical Distribution

Throughout the study period, a seasonal monthly pattern in salmonellosis frequency was observed with mean annual notification rates increasing from spring through the summer months, peaking in August, and then gradually decreasing during autumn (Table 5).

**Figure 2:**
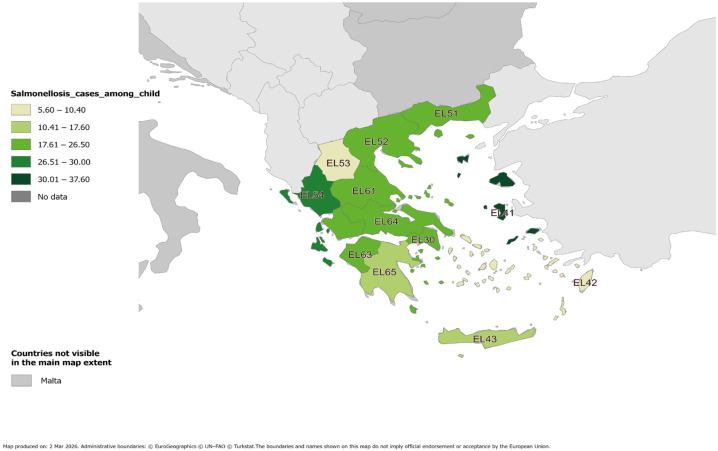
Mean annual notification rate of salmonellosis cases among children 0–14 years of age by region, MNS, Greece, 2005–2024. Region labels (EL codes) correspond to NUTS-2 geographic classification codes used by Eurostat.

Among children aged < 15 years during 2005–2024, the highest mean annual notification rate was observed in the North Aegean Islands (37.7 cases per 100,000 population), while the lowest was observed in Western Macedonia (5.6 cases per 100,000 population) (Supplementary Table S1). These data are presented geographically on the map shown in Figure 2.

Age-stratified analysis showed that in the 0–4-year age group, the highest and lowest mean annual notification rates were observed in the North Aegean Islands (66.4 cases per 100,000 population) and Western Macedonia (11.5 cases per 100,000 population), respectively. In the 5–9-year age group, the highest and lowest mean annual notification rates were observed in the North Aegean Islands (31.0 cases per 100,000 population) and Western Macedonia (3.4 cases per 100,000 population), respectively. In the 10–14-year age group, the highest and lowest mean annual notification rates were observed in the North Aegean Islands (15.6 cases per 100,000 population) and Western Macedonia (2.8 cases per 100,000 population), respectively. Detailed data are presented in Supplementary Table S2, and geographically on the maps shown in Supplementary Figures S2–S4.

### 3.6. Serovar Distribution

During the period 2005–2024, 2823 *Salmonella* isolates (38%) were serotyped at the NSSRC. The most frequently recorded serovars among children aged 0–14 years over the 20-year surveillance period were *S.* Enteritidis (54%), *S.* Typhimurium (13%), *Salmonella* Bovismorbificans (*S.* Bovismorbificans) (4%), monophasic *Salmonella* Typhimurium (monophasic *S.* Typhimurium) (4%), *Salmonella* Oranienburg *(S.* Oranienburg) (2%), and *Salmonella enterica* subsp. *salamae* (II) (1%). The percentage distribution of *Salmonella* serovars among children aged 0–14 years by year during the surveillance period is presented in Table 6.

Age-stratified analysis showed that the most frequently identified serovars during the 2005–2024 surveillance period across all age groups were *S.* Enteritidis and *S.* Typhimurium. Similarly, these two serovars predominate in all age groups and among children with invasive infection. These data are presented in Supplementary Table S3.

## 4. Discussion

In summary, our study shows that during 2005–2024, salmonellosis notification rates among children under 15 years of age declined until 2021, followed by a moderate increase thereafter. The higher burden observed among children aged 0–4 years aligns with established epidemiological patterns of enteric infections in early childhood and may reflect increased biological susceptibility and greater likelihood of healthcare-seeking behavior. The pronounced summer peak indicates a stable and recurrent transmission pattern, highlighting the importance of temporally targeted prevention measures. Geographical heterogeneity was also observed, with higher mean annual rates in the North Aegean Islands and lower rates in Western Macedonia. This may reflect either true regional variation or differences in case detection, healthcare access, laboratory capacity, etc.; however, in the absence of detailed contextual data, these differences cannot be attributed to specific underlying factors. Finally, the sustained predominance of *S.* Enteritidis and *S.* Typhimurium suggests continued circulation of these serovars despite long-standing control measures.

The high proportion of pediatric cases could be attributed either to a genuinely higher burden among children or to increased diagnostic testing and healthcare-seeking behavior in this population [19,20]. Analysis of hospitalization data revealed a high frequency of hospitalizations across all age groups, whereas hospitalizations due to severe (invasive) infections remained low. These findings along with the rarely observed mortality underscore that the disease is generally mild, although severe cases may occur [29]. This discrepancy suggests that hospitalization in passive surveillance systems should be interpreted cautiously when used as an indicator of clinical severity. Disease incidence was higher among children aged 0–4 years, a finding consistent with reports from other countries [33] and compatible with increased vulnerability in early childhood. Factors such as dietary transition, introduction of new foods including eggs, evolving hygiene practices, and developmental behaviors likely contribute to exposure risk during this period of life.

Data analysis showed declining salmonellosis notification rates in Greece up to 2021, followed by an increase. However, given the descriptive non-inferential nature of this surveillance analysis, formal time-series modeling or regression-based trend testing was not performed. Globally, surveillance data demonstrated a strong declining trend of human salmonellosis until 2015, with stabilization thereafter until 2020 [34,35,36,37,38]. During the pandemic period, incidence remained stable during the pandemic and increased thereafter [39,40]. Within the EU/EEA, statistically significant declining trends were recorded up to the pandemic years in several countries including Greece [33,41,42,43,44]. These parallel temporal patterns suggest that the observed decline was not unique to Greece but part of a broader European trend. It should also be considered that the observed temporal patterns may have been partially influenced by changes in the surveillance system over time. These changes may have affected case ascertainment and reporting completeness. Public health measures implemented during the pandemic, such as travel restrictions, reduced social contact, restaurant closures, and intensified hygiene practices, may have affected both exposure opportunities and case detection [45]. The increase observed after 2021 may reflect a combination of factors. This may include either a true increase in exposure as social mixing, travel, and food-service activity resumed, or a rebound in healthcare-seeking behavior, diagnostic testing, and surveillance performance following pandemic-related disruptions. However, given the descriptive nature of the dataset and study, causal conclusions cannot be drawn. Outside the EU/EEA, similar long-term declines followed by post-pandemic increases were observed in the United States [40,46,47] and in the United Kingdom [48,49,50], further supporting the interpretation that both structural control measures and contextual surveillance factors contributed to the observed trajectory.

During the study period, the most frequently identified serovars among children aged 0–14 years were *S.* Enteritidis, *S.* Typhimurium, monophasic *S.* Typhimurium, *S.* Bovismorbificans, *S.* Oranienburg, and *S. enterica* subsp. *salamae* (II). Following age stratification, the same serovars predominated. Monitoring and control programs were developed within the European Union (EU) [51] and implemented in Greece since 2007 to reduce targeted *Salmonella* serovars in poultry populations. These programs contributed to reductions in *S.* Enteritidis and *S.* Typhimurium prevalence in poultry and to declines in human salmonellosis at the European level [25,50,52]; however, these serovars remain dominant among human cases in Europe [40]. From a One Health perspective, the persistence of *S.* Enteritidis and *S.* Typhimurium in humans, along with their detection in poultry and other animal sources, underscores the significance of integrated One Health surveillance that links veterinary and human laboratory data, including AMR data, to better understand transmission dynamics of Salmonella serovars.

Our analysis also highlighted a seasonal pattern in disease occurrence in Greece. Notification rates increased from spring, peaked in August, and declined thereafter. Seasonality may be partially explained by increasing temperatures from spring through summer, along with increased outdoor activities and social interactions. In addition, higher environmental temperatures favor the growth of *Salmonella*, leading to higher pathogen concentrations in the food chain during warmer months [53,54,55,56]. This finding is consistent with findings from other European countries [28,39]. Similar late-summer peaks have been documented in Canada [57] and the United States [58], whereas in the Southern Hemisphere, peak incidence occurs during the warmest month of the year [59]. Reports from the United Kingdom [60], Italy [61], and Scandinavian countries [62] describe comparable seasonal patterns. The stability of this seasonal curve over two decades suggests that climatic and behavioral factors continue to play a central role in transmission dynamics.

The geographical distribution of pediatric cases showed the highest mean annual notification rates in the North Aegean Islands and the lowest in Western Macedonia. Although no specific explanatory factor could be identified from the available dataset, the persistence of this pattern across age groups indicates that regional variation is consistent over time. In the absence of detailed outbreak or exposure data, interpretation should remain cautious, and enhanced regional surveillance may help clarify underlying drivers.

Several limitations should be acknowledged. Missing data affected multiple database variables, and the absence of information on suspected food items precluded identification of specific exposures. Reporting delays and incomplete forms reduced the completeness of clinical data. The surveillance system does not permit systematic case follow-up, limiting outcome assessment. In addition, the lack of antimicrobial susceptibility data restricted analysis of severe and hospitalized cases. The high hospitalization proportions likely reflected characteristics of passive, clinician-based surveillance and hospital-dominated reporting. Thus, milder cases managed in outpatient settings may have been underrepresented. Therefore, hospitalization proportions should not be interpreted as a direct proxy of clinical severity in the population. Finally, the absence of outbreak investigation data prevented linkage of cases to specific sources or vehicles of transmission. Nevertheless, the extended observation period and nationwide coverage provide a comprehensive overview of pediatric salmonellosis trends in Greece.

The findings of this study have several public health implications despite limitations. The consistently higher burden among children aged 0–4 years supports the need for targeted parental education on safe food handling and hygiene practices in domestic kitchens, particularly during periods of dietary transition. The stable and pronounced summer peak indicates that prevention efforts and food safety messaging should be intensified prior to and during high-temperature months. Increased clinical awareness among pediatric healthcare providers, especially during the summer season, may facilitate timely diagnosis and appropriate parental counseling. Strengthening surveillance completeness, improving laboratory data integration, and expanding serotyping coverage would further enhance monitoring capacity and inform control strategies.

## 5. Conclusions

Despite advances in understanding *Salmonella* pathogenesis, improvements in infection control strategies, and enhanced sanitary conditions, the burden of non-typhoidal salmonellosis in children remains substantial and, in some regions, continues to increase. This persistence may also relate to the emergence of antimicrobial resistance [63]. Continued investigation of epidemiological patterns, clinical characteristics, exposures, and antimicrobial susceptibility profiles in the pediatric population is essential to enhance surveillance capacity and inform public health planning and prevention strategies. In parallel, strengthening surveillance systems and implementing targeted, seasonally focused prevention strategies remain critical for reducing disease burden.

## Figures and Tables

**Table 1:** Salmonellosis cases, annual case notification rates, number of hospitalized salmonellosis cases, annual hospitalized case notification rates, number of severe hospitalized salmonellosis cases and severe hospitalization notification rates, among children 0–14 years old, per year of notification, MNS, Greece, 2005–2024.

Year of Notification	Total Number of Cases	Annual Notification Rate per 100,000 Population	Total Number of Hospitalized Cases	Annual Hospitalized Notification Rate per 100,000 Population	Number of Hospitalized Cases with Invasive Infection	Annual Notification Rate of Hospitalized Cases with Invasive Infection per 100,000 Population
2005	676	40.9	599	36.2	27	1.6
2006	486	29.6	457	27.9	11	0.7
2007	388	23.8	348	21.4	10	0.6
2008	481	29.7	431	26.6	8	0.5
2009	261	16.1	241	14.8	7	0.4
2010	187	11.5	169	10.4	5	0.3
2011	313	19.2	285	17.5	12	0.7
2012	269	16.5	249	15.3	11	0.4
2013	259	16.0	219	13.6	25	1.5
2014	227	14.2	195	12.2	17	1.1
2015	307	19.5	259	16.4	16	1.0
2016	446	28.7	378	24.3	15	1.0
2017	403	25.9	350	22.5	17	1.1
2018	394	25.5	351	22.7	9	0.6
2019	360	23.4	306	19.9	17	1.1
2020	221	14.5	186	12.2	20	1.3
2021	165	10.9	143	9.5	11	0.7
2022	357	25.3	298	21.1	12	0.8
2023	434	31.3	355	25.6	11	0.8
2024	520	38.3	442	32.5	11	0.8
Total	7154					
Mean	357.7	23.0	322.0	19.0	13.6	0.9
SD	142.7	8.8	122.9	7.7	5.7	0.4

**Table 2:** Annual and average monthly mean notification salmonellosis case rates among children 0–14 years old, per age group, per year of notification, MNS, Greece, 2005–2024.

	Annual Notification Rates			Average Monthly Notification Rates		
Year of Notification	0–4 Years Old	5–9 Years Old	10–14 Years Old	0–4 Years Old	5–9 Years Old	10–14 Years Old
2005	78.7	30.8	15.6	6.6	2.6	1.3
2006	53.7	25.1	11.6	4.5	2.1	1.0
2007	41.9	22.1	8.1	3.5	1.8	0.7
2008	51.7	24.0	13.7	4.3	2.0	1.1
2009	27.2	15.7	5.3	2.3	1.3	0.4
2010	22.4	8.1	3.7	1.9	0.7	0.3
2011	35.4	15.8	5.9	2.9	1.3	0.5
2012	35.6	9.2	4.3	3.0	0.8	0.4
2013	32.0	11.2	4.7	3.0	0.9	0.4
2014	28.8	10.9	3.2	2.4	0.9	0.3
2015	36.6	16.6	6.2	3.1	1.4	0.5
2016	55.0	20.6	13.1	4.6	1.7	1.1
2017	56.1	17.7	7.3	4.7	1.5	0.6
2018	45.3	22.1	11.4	3.8	1.9	1.0
2019	44.6	18.2	10.3	3.8	1.5	0.9
2020	31.4	10.0	4.5	2.6	0.8	0.4
2021	23.8	6.0	4.8	2.0	0.5	0.4
2022	111.9	33.2	14.8	9.3	2.8	1.2
2023	113.6	50.8	29.1	9.5	4.5	2.4
2024	72.4	30.7	18.5	6.0	2.7	1.5
Mean	49.9	19.9	9.8	4.2	1.7	0.8
SD	26.2	10.7	6.4	2.2	1.0	0.5

**Table 3:** Salmonellosis clinical manifestations among children 0–14 years old, per age group, MNS, Greece, 2005–2024.

	Symptoms					
	Enteritis	Enteric Fever	Dysentery	Sepsis	Uremia	Other
0–4 years old						
total	3305 (81%)	641 (16%)	328 (8%)	57 (1%)	7 (0.2%)	431 (12%)
male	1763 (53%)	347 (54%)	179 (55%)	28 (49%)	3 (43%)	246 (57%)
female	1542 (47%)	294 (46%)	149 (45%)	29 (51%)	4 (57%)	185 (43%)
5–9 years old						
total	1441 (81%)	342 (19%)	169 (10%)	10 (1%)	1 (0.1%)	168 (12%)
male	780 (54%)	180 (53%)	97 (57%)	6 (60%)	0 (0%)	86 (51%)
female	661 (46%)	192 (47%)	72 (43%)	4 (40%)	1 (100%)	82 (49%)
10–14 years old						
total	736 (81%)	164 (18%)	86 (9%)	2 (0.2%)	1 (0.1%)	97 (10%)
male	431 (59%)	98 (60%)	52 (60%)	1 (50%)	0	54 (56%)
female	305 (41%)	66 (40%)	34 (40%)	1 (50%)	1 (100%)	43 (44%)

**Table 4:** Salmonellosis cases among children 0–14 years old per year of notification, per age group, MNS, Greece, 2005–2024.

	Total Number of Salmonellosis Cases					
	0–4 Years Old		5–9 Years Old		10–14 Years Old	
Year of Notification	Total Number of Cases	Total Number of Hospitalized Cases, of Which Severe (%)	Total Number of Cases	Total Number of Hospitalized Cases, of Which Severe (%)	Total Number of Cases	Total Number of Hospitalized Cases, of Which Severe (%)
2005	419	368 (6%)	166	148 (2%)	91	83 (4%)
2006	285	267 (4%)	135	126 (0%)	66	64 (1%)
2007	224	197 (4%)	119	112 (3%)	45	39 (0%)
2008	277	245 (3%)	129	117 (2%)	75	69 (0%)
2009	148	134 (4%)	84	79 (0%)	29	28 (4%)
2010	124	111 (5%)	43	38 (0%)	20	20 (0%)
2011	197	177 (6%)	84	78 (0%)	32	30 (3%)
2012	197	184 (6%)	49	44 (0%)	23	21 (0%)
2013	174	148 (12%)	60	51 (13%)	25	20 (10%)
2014	151	129 (12%)	59	51 (2%)	17	15 (0%)
2015	183	158 (5%)	91	77 (9%)	33	24 (4%)
2016	264	218 (5%)	113	100 (3%)	69	60 (2%)
2017	267	230 (6%)	97	87 (4%)	39	33 (0%)
2018	213	189 (2%)	120	104 (4%)	61	58 (2%)
2019	208	182 (6%)	96	79 (1%)	56	45 (6%)
2020	145	122 (13%)	51	42 (7%)	25	22 (5%)
2021	108	91 (9%)	30	25 (12%)	27	27 (0%)
2022	237	201 (6%)	79	65 (0%)	41	32 (0%)
2023	236	195 (3%)	119	96 (4%)	79	64 (1%)
2024	287	242 (1%)	138	117 (2%)	95	83 (5%)
Total	4344	3788 (5%)	1862	1636 (3%)	948	837 (2%)

**Table 5:** Mean notification rates of salmonellosis cases among children 0–14 years of age, per month of notification, MNS, Greece, 2005–2024.

Month of Notification	Total Number of Cases	Mean Monthly Notification Rate (Cases/100,000 Population)
January	270	2.9
February	183	2.0
March	223	2.4
April	243	2.6
May	464	5.2
June	636	7.4
July	1016	11.6
August	1335	16.0
September	1125	13.6
October	774	9.1
November	565	6.3
December	320	3.6

**Table 6:** Frequency distribution of most common and other serovars per age group, among children < 15 years old, MNS and NSSRC, Greece, 2005–2024.

Year of Notification	*S.* Enteritidis n (%)	*S.* Typhimurium n (%)	Monophasic *S.* Typhimurium n (%)	*S.* Bovismorbificans n (%)	*S.* Oranienburg n (%)	*S. enterica* subsp. *salamae* (II) n (%)	Other Serovars n (%)	Total n (%)
2005	252 (48.6)	28 (5.4)	0	0	0	10 (1.9)	228 (44.0)	518 (100)
2006	154 (39.3)	13 (3.3)	0	0	0	4 (1.0)	221 (56.4)	392 (100)
2007	126 (37.6)	10 (3.0)	0	0	0	2 (0.6)	197 (58.8)	335 (100)
2008	123 (34.7)	8 (2.3)	0	0	0	3 (0.8)	220 (62.1)	354 (100)
2009	40 (23.5)	3 (1.8)	0	1 (0.6)	0	2 (1.2)	124 (72.9)	170 (100)
2010	18 (10.5)	2 (1.2)	0	0	0	1 (0.6)	151 (87.8)	172 (100)
2011	22 (8.9)	15 (6.1)	0	0	0	1 (0.4)	209 (84.6)	247 (100)
2012	25 (9.7)	25 (9.7)	0	0	0	1 (0.4)	208 (80.3)	259 (100)
2013	43 (17.8)	40 (16.5)	9 (3.7)	6 (2.5)	1 (0.4)	2 (0.8)	141 (58.3)	242 (100)
2014	39 (17.0)	30 (13.1)	3 (1.3)	12 (5.2)	3 (1.3)	1 (0.4)	141 (61.6)	229 (100)
2015	106 (36.4)	24 (8.2)	1 (0.3)	19 (6.5)	13 (4.5)	0	128 (44.0)	291 (100)
2016	124 (30.1)	17 (4.1)	14 (3.4)	7 (1.7)	6 (1.5)	0	244 (59.2)	412 (100)
2017	67 (18.9)	30 (8.5)	54 (15.2)	4 (1.1)	5 (1.4)	0	195 (54.9)	355 (100)
2018	64 (20.9)	29 (9.5)	7 (2.3)	2 (0.7)	1 (0.3)	2 (0.7)	201 (65.7)	306 (100)
2019	49 (15.4)	7 (2.2)	8 (2.5)	0	5 (1.6)	1 (0.3)	248 (78.0)	318 (100)
2020	26 (14.7)	10 (5.6)	0	6 (3.4)	5 (2.8)	0	130 (73.4)	177 (100)
2021	29 (24.0)	15 (12.4)	0	10 (8.3)	7 (5.8)	2 (1.7)	58 (47.9)	121 (100)
2022	31 (8.6)	7 (1.9)	0	11 (3.0)	0	4 (1.1)	309 (85.4)	362 (100)
2023	102 (22.5)	25 (5.5)	5 (1.1)	8 (1.8)	4 (0.9)	1 (0.2)	308 (68.0)	453 (100)
2024	88 (16.2)	37 (6.8)	4 (0.7)	35 (6.4)	8 (1.5)	2 (0.4)	371 (69.4)	545 (100)

## Data Availability

Data are unavailable due to privacy and ethical restrictions.

## Supplementary Materials

The following supporting information can be downloaded at: https://www.mdpi.com/article/10.3390/microorganisms14040743/s1, Figure S1: Time distribution of salmonellosis notification rates per age group, MNS, Greece, 2005–2024, Table S1: Total mean annual case notification rates and mean annual notification rates among children 0–14 years old, per region, MNS, Greece, 2005–2024, Table S2: Mean annual case notification rates among children 0–14 years old, per age group, per region, MNS, Greece, 2005–2024, Figure S2: Mean annual notification rate of salmonellosis cases among children 0–4 years of age by region, MNS, Greece, 2005–2024. Region labels (EL codes) correspond to NUTS-2 geographic classification codes used by Eurostat., Figure S3: Mean annual notification rate of salmonellosis cases among children 5–9 years of age by region, MNS, Greece, 2005–2024. Region labels (EL codes) correspond to NUTS-2 geographic classification codes used by Eurostat., Figure S4: Mean annual notification rate of salmonellosis cases among children 10–14 years of age by region, MNS, Greece, 2005–2024. Region labels (EL codes) correspond to NUTS-2 geographic classification codes used by Eurostat., Table S3: Frequency distribution of most common and other serovars per age group, among children <15 years old (and among those with invasive infection), MNS and NSSRC, Greece, 2005–2024.

## Abbreviations

The following abbreviations are used in this manuscript:

AMR	Antimicrobial Resistance
CI	Confidence Interval
COVID-19	Coronavirus Disease 2019
DESIID	Directorate of Epidemiological Surveillance and Interventions for Infectious Diseases
ECDC	European Centre for Disease Prevention and Control
ELSTAT	Hellenic Statistical Authority
EMMa	European Monitoring Map Maker
EU	European Union
EU/EEA	European Union/European Economic Area
GDPR	General Data Protection Regulation
HIV	Human Immunodeficiency Virus
IQR	Interquartile Range
MNS	Mandatory Notification System
NSSRC	National Salmonella–Shigella Reference Centre
EODY	National Public Health Organization
*S.* Bovismorbificans	*Salmonella* Bovismorbificans
*S.* Enteritidis	*Salmonella* Enteritidis
*S.* Oranienburg	*Salmonella* Oranienburg
*S.* Typhimurium	*Salmonella* Typhimurium
spp.	Species (plural)
TESSy	The European Surveillance System
