# Supplementary material for: Salmonellosis Among Children Aged 0–14 Years in Greece over the Period 2005–2024: Descriptive Analysis of Surveillance Data from the Mandatory Notification System
Source: Microorganisms. 2026 Mar 26;14(4):743. doi: 10.3390/microorganisms14040743 (PMC13118311; doi:10.3390/microorganisms14040743)
Supplement: Supplementary file 1 [file microorganisms-14-00743-s001.zip › Table S1.pdf]

Table S1. Total mean annual case notification rates and mean annual notification rates among children 0-14 years old, per region, MNS, Greece, 2005-2024

Region	Total Population	Total cases	Mean annual notification rate (cases/100,000 population)
Attika	10,930,431	2,569	1.2
Peloponnese	1,596,757	239	0.7
Central Greece	1,568,016	365	1.2
Thessaly	2,090,887	519	1.2
Epirus	892,813	268	1.5
Western Macedonia	781,839	44	0.3
Central Macedonia*	5,529,136	1,286	1.2
Eastern Macedonia – Thrace	1,752,173	464	1.3
Western Greece	1,976,110	466	1.2
Crete	2,067,874	363	0.9
Ionian Islands	595,236	176	1.5
Northern Aegean Islands	640,033	241	1.9
Southern Aegean Islands	1,138,385	118	0.5

* The Administration of Mouth Athos is included
