# Supplementary material for: Salmonellosis Among Children Aged 0–14 Years in Greece over the Period 2005–2024: Descriptive Analysis of Surveillance Data from the Mandatory Notification System
Source: Microorganisms. 2026 Mar 26;14(4):743. doi: 10.3390/microorganisms14040743 (PMC13118311; doi:10.3390/microorganisms14040743)
Supplement: Supplementary file 1 [file microorganisms-14-00743-s001.zip › Table S2.pdf]

Table S2. Mean annual case notification rates among children 0-14 years old, per age group, per region, MNS, Greece, 2005-2024.

	**0-4 years old**		**5-9 years old**		**10-14 years old**	
**Region**	**Total Population**	**Mean annual notification rate (cases/100,000 population)**	**Total Population**	**Mean annual notification rate (cases/100,000 population)**	**Total Population**	**Mean annual notification rate (cases/100,000 population)**
Attika	3,631,112	2.2	3,644,604	0.9	3,654,715	0.5
Peloponnese	491,600	1.5	540,579	0.5	564,578	0.3
Central Greece	475,488	2.5	534,717	0.8	557,811	0.4
Thessaly	644,548	2.4	700,322	1.1	746,017	0.4
Epirus	275,024	2.9	297,301	1.1	320,487	0.7
Western Macedonia	233,973	0.6	262,799	0.2	285,067	0.1
Central Macedonia*	1,716,921	2.1	1,857,528	1.0	1,954,686	0.5
Eastern Macedonia – Thrace	541,514	2.2	586,836	1.3	623,824	0.6
Western Greece	604,697	2.7	660,308	0.6	711,105	0.4
Crete	677,925	1.7	692,291	0.7	697,658	0.3
Ionian Islands	191,751	3.0	200,068	0.8	203,417	0.7
Northern Aegean Islands	212,366	3.3	216,382	1.5	211,284	0.8
Southern Aegean Islands	367,181	1.0	382,266	0.4	388,938	0.2

* The Administration of Mouth Athos is included
