# Supplementary material for: Salmonellosis Among Children Aged 0–14 Years in Greece over the Period 2005–2024: Descriptive Analysis of Surveillance Data from the Mandatory Notification System
Source: Microorganisms. 2026 Mar 26;14(4):743. doi: 10.3390/microorganisms14040743 (PMC13118311; doi:10.3390/microorganisms14040743)
Supplement: Supplementary file 1 [file microorganisms-14-00743-s001.zip › Table S3.pdf]

Table S3. Frequency distribution of most common and other serovars per age group, among children <15 years old (and among those with invasive infection), MNS and NSSRC, Greece, 2005 – 2024.

Age group	% Identified serovars (with invasive infection)					
	S. Enteritidis	*S.* Typhimurium	Monophasic *S.* Typhimurium	*S.* Bovismorbificans	*S.* Oranienburg	*S.* arizona
0-4 years old	22.1 (19.2)	6.6 (6.9)	1.6 (0.7)	2.5 (2.7)	1.1 (4.8)	0.7 (0)
5-9 years old	28.4 (19.4)	5.8 (9.7)	1.8 (6.5)	1.2 (3.2)	0.6 (3.2)	0.6 (0)
10-14 years old	27.5 (20.0)	3.8 (6.7)	1.7 (0)	0.9 (0)	0.7 (6.7)	0.6 (0)
