# Supplementary figures and images for: Salmonellosis Among Children Aged 0–14 Years in Greece over the Period 2005–2024: Descriptive Analysis of Surveillance Data from the Mandatory Notification System
Source: Microorganisms. 2026 Mar 26;14(4):743. doi: 10.3390/microorganisms14040743 (PMC13118311; doi:10.3390/microorganisms14040743)

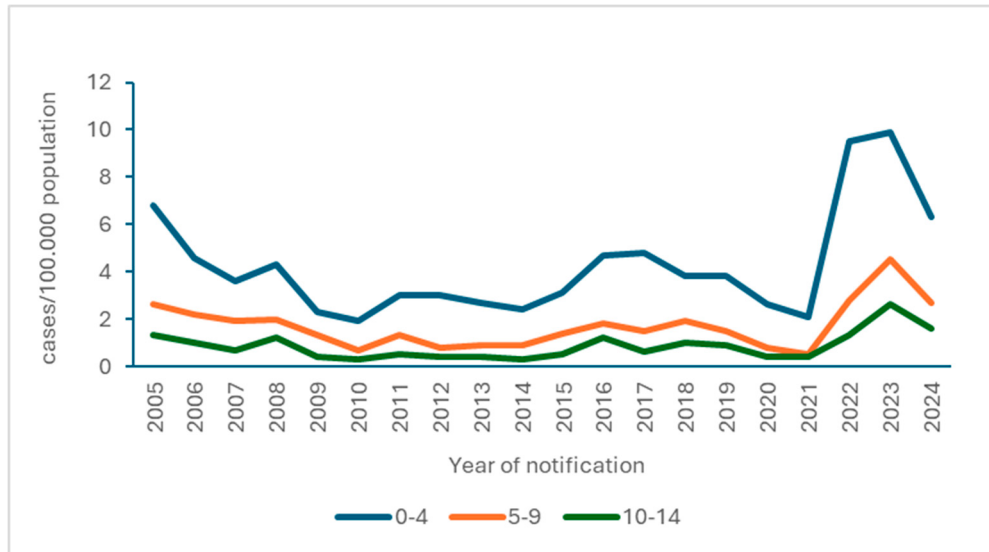

**Figure S1.** Time distribution of salmonellosis notification rates per age group, MNS, Greece, 2005–2024

Supplement: Supplementary file 1 [file microorganisms-14-00743-s001.zip › Figure S1.pdf]
