# Supplementary material for: Salmonellosis Among Children Aged 0–14 Years in Greece over the Period 2005–2024: Descriptive Analysis of Surveillance Data from the Mandatory Notification System
Source: Microorganisms. 2026 Mar 26;14(4):743. doi: 10.3390/microorganisms14040743 (PMC13118311; doi:10.3390/microorganisms14040743)
Supplement: Supplementary file 1 [file Figure S4.pdf › Figure S4.pdf]

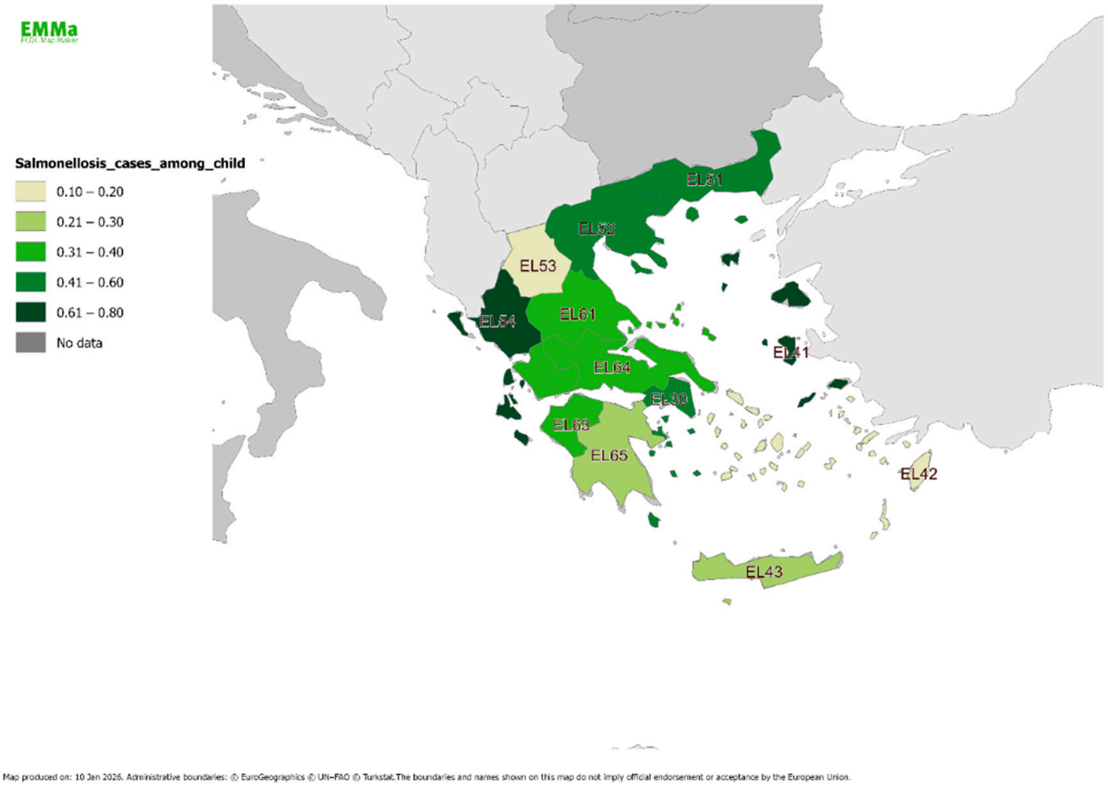

Figure S4. Mean annual notification rate of salmonellosis cases among children 10-14 years of age by region, MNS, Greece, 2005-2024. Region labels (EL codes) correspond to NUTS-2 geographic classification codes used by Eurostat.
